# Melatonin Inhibits the Migration of Human Lung Adenocarcinoma A549 Cell Lines Involving JNK/MAPK Pathway

**DOI:** 10.1371/journal.pone.0101132

**Published:** 2014-07-03

**Authors:** Qiaoyun Zhou, Shuyu Gui, Qing Zhou, Yuan Wang

**Affiliations:** 1 Department of Respiratory Medicine, the First Affiliated Hospital, Anhui Medical University, Hefei, Anhui, China; 2 Laboratory of Molecular Biology and Department of Biochemistry, Anhui Medical University, Hefei, Anhui, China; 3 Key Laboratory of Gene Research of Anhui Province, Anhui Medical University, Hefei, Anhui, China; University of Alabama at Birmingham, United States of America

## Abstract

**Objective:**

Melatonin, an indolamine produced and secreted predominately by the pineal gland, exhibits a variety of physiological functions, possesses antioxidant and antitumor properties. But, the mechanisms for the anti-cancer effects are unknown. The present study explored the effects of melatonin on the migration of human lung adenocarcinoma A549 cells and its mechanism.

**Methods:**

MTT assay was employed to measure the viability of A549 cells treated with different concentrations of melatonin. The effect of melatonin on the migration of A549 cells was analyzed by wound healing assay. Occludin location was observed by immunofluorescence. The expression of occludin, osteopontin (OPN), myosin light chain kinase (MLCK) and phosphorylation of myosin light chain (MLC), JNK were detected by western blots.

**Results:**

After A549 cells were treated with melatonin, the viability and migration of the cells were inhibited significantly. The relative migration rate of A549 cells treated with melatonin was only about 20% at 24 h. The expression level of OPN, MLCK and phosphorylation of MLC of A549 cells were reduced, while the expression of occludin was conversely elevated, and occludin located on the cell surface was obviously increased. The phosphorylation status of JNK in A549 cells was also reduced when cells were treated by melatonin.

**Conclusions:**

Melatonin significantly inhibits the migration of A549 cells, and this may be associated with the down-regulation of the expression of OPN, MLCK, phosphorylation of MLC, and up-regulation of the expression of occludin involving JNK/MAPK pathway.

## Introduction

Lung cancer is the most common cancer and the leading cause of cancer deaths worldwide. The prognosis of patients can be improved through effective treatment, but the 5-year survival rate of the patients with advanced lung cancer is only about 16% [1]. Various novel therapeutic strategies currently under consideration as the clinical use of cytotoxic drugs is limited due to intrinsic or acquired resistant and toxicity [2]. The majority of patients with lung cancer presents with locally advanced inoperable or metastatic disease [3].

Cell migration is a biological process that contributes crucially to a variety of physiological, wound healing and the inflammatory reaction. Moreover, cell migration is also responsible for the malignance of cancer disease as it allows tumor cells to invade the surrounding tissues, thereby forming metastases [4].

Recent studies demonstrate that many proteins, such as myosin light-chain kinase (MLCK), osteopontin (OPN), play a critical role in non-muscle cell protrusion, contraction, and migration [5]–[6]. MLCK is a key Ca2+/Calmodulin (CaM)-dmependent effector that is responsible for smooth muscle cell and non-muscle cell migration via phosphorylation of Ser19, Thr18 on myosin light chains (MLC), an event that facilitates myosin interaction with actin filaments [7]. MLCK expression reduction via antisense techniques is lead to rounding fibroblast cell, decreasing proliferation and attenuating chemoattractant-stimulated cell locomotion [8]. OPN undergoes extensive posttranslational modification, including phosphorylation, glycosylation and cleavage, resulting in molecular mass variants ranging from 25 to 75 kDa [9]. There is evidence suggesting that multiple signals may function in OPN-mediated tumor cell migration as inhibitors to phospholipase C/protein kinase C (PLC/PKC), mitogen activated protein kinase (MAPK), and PI3K could decrease OPN-induced migration [6].

Tumor cells, particularly in those cancers that manifest high metastatic potential, often exhibit loss of tight junctions (TJ). Occludin is a transmembrane protein of epithelial TJs, therefore its structure is relatively well characterized [10]–[12]. Down-regulation of specific TJ proteins has been shown to correlate with the staging, invasiveness and metastasis potential of epithelial cancers [13]–[15]. MAPK signaling pathway is able to modulate TJ paracellular transport by up-or down-regulating the expression of several TJ proteins and hence altering the molecular composition within TJ complexes [16].

These observations directly implicate that MLCK, OPN, occludin in the signaling pathways controls the non-muscle cell motility. However, the exact mechanistics during cancer cell migration remain poorly understood.

MAPK pathways play pivotal roles in cell proliferation, differentiation, and survival [17]. The closely related MAPK pathways are regulated through a series of phosphorylation steps in a three-component module: MAPKs are activated by MAPK kinases (MAPKK) on dual residues of threonine and tyrosine, and MAPKKs are in turn phosphorylated by MAPKK kinases (MAPKKK) on dual residues of serine/threonine. MAPKs have been divided into three main groups: the extracellular-regulated kinases 1/2(ERK1/2 or MAPK p44/42), MAP 38, and the c-jun-N-terminal kinases (JNK) [18]. It has been reported that JNK is constitutively activated in several tumor cell lines and that the transforming actions of several oncogenes have been reported to be JNK dependent (based on dominant-negative approaches) [19]. Recently, more and more evidences indicate that JNK substrates, especially the non-nuclear proteins, also have wide-ranging functional roles in cell migration, axonal guidance, neurite formation and outgrowth, brain development, dendritic architecture and regeneration of nerve fibers after injury [20].

Melatonin is an indole bioactivator mainly secreted by the pineal gland. It has a wide range of reported biologic effects including antioxidative [21]–[26], anti-inflammatory and antitumor activities [27]–[34] and has generated considerable interest as a pharmaceutical compound with a wide range of therapeutic activities. Melatonin has also been shown to possess chemotherapeutic potential in human cancers and to be capable of modulating several signal transduction pathways associated with cell survival, proliferation, apoptosis and invasion [35]–[39]. Recent studies have reported that melatonin can inhibit tumor invasion through increasing adhesion by elevating E-cadherin and β1-integrin expression [40] or modulating microfilament [41]–[42], and decreasing matrix metalloproteinases (MMPs) production [43]. Anti-invasion effect of melatonin has been shown in human mammary epithelial cancer MCF-7 cells [40]–[41], [43] and MDCK cells [42].

However, it is unknown whether melatonin affects the migration and invasion of A549 cells via OPN, occludin, as well as MLCK and through which pathway. Therefore, the present study was undertaken to investigate the effect of melatonin on the migration and invasion of A549 cells. In addition, we also assess the expression of OPN, occludin, MLCK, and the function of JNK MAPK signal transduction pathway.

## Materials and Methods

### Cell culture

Human lung adenocarcinoma cell line (A549) was purchased from ATCC and cultured in Dulbecco’s modified Eagle’s medium (DMEM) with 10% fetal bovine serum (TBD Science, Tianjin, China), 1 mmol/L glutamine, 100 U/mL penicillin and10 mg/mL streptomycin (Ameresco, USA), at 37°C, 5% CO_2_.

### Reagents

Melatonin was provided by School of Pharmacy, Anhui Medical University (Anhui, China). Dimethyl Sulfoxide (DMSO) was obtained from sigma Chemical (USA). Dulbeccos modified Eagles medium (DMEM) was purchased from Gibco BRL life Technologies (USA). Phorbol-12-myristate-13-acetate (PMA) and SP600125 were obtained from Cayman Chemical (USA). Primary antibodies (anti-OPN, anti-occludin, anti-MLCK, anti-pMLC, anti-MLC, anti-pJNK, anti-JNK, anti-β-actin) were purchased from Santa Cruz Biotechnology (USA). All secondary antibodies were purchased from Millipore (USA). ECL reagent and BCA kit were purchased from quantitative Pierce Company.

### Cell viability assay

Cell viability was measured using the MTT assay. A549 cells (4.5×104 cells/well) were seeded into 96-well plates and cultured. The cells were treated with different concentrations of melatonin (0.1, 0.5, 0.75, 1.0, 2.5, 5.0 mmol/L), then incubated with MTT solution for 4 h. Finally, the cells were exposed to an MTT-formazan dissolving solution (DMSO) for 30 minutes. The optical density (OD) was measured using an absorbance microplate reader (Bio-Tek, ELX800) at a wavelength of 490 nm. The cell viability was expressed as a percentage of the OD value of the control cultures. For the A549 cells, the data were taken from one experiment with 4 replicates. IC_50_ was determined using a sigmoidal equilibrium model regression using XLfit version 4.3.2 (ID Business Solutions Ltd.) and is defined as the concentration of melatonin required for a 50% reduction in growth/viability.

### Wound healing assay

Migration of A549 cells was measured using the wound-healing assay in vitro. Cells were seeded into 12-well plates and grown to 100% confluence. Wounds were created by scraping monolayer cells with a sterile pipette tip. At 0, 12, 24 h after the creation of wounds, cells were observed with 10×objective in an Olympus (Olympus Corporation, Tokyo, Japan) photomicroscope. Images were acquired with a Nikon (Tokyo, Japan) color digital camera. Wound distances were measured at each time point and expressed as the average percent of wound closure by comparing the zero time.

### Immunofluorescence assay

A549 cells (0.5×10^4^ cells/well) were seeded into 96-well plates with sterile aseptic cover glasses and cultured. Cells were treated with 0.1 mmol/L and 2.0 mmol/L concentration melatonin for 7 d. DMSO was added in the control group. The nutrient solution with melatonin was changed every day. After treatment, the cells were washed and fixed with 4% paraformaldehyde for 20 minutes at room temperature, then washed and blocked with blocking buffer (5% nonfat dry milk in PBS) for 2 h at room temperature. The cover glass with A549 cells was incubated with goat anti-human occludin (1∶50) primary antibody overnight at 4°C. Cells were washed and incubated with donkey anti-goat IgG-FITC (1∶200), then washed and mounted with aqueous-based anti-fade mounting medium. Images of stained cells were captured using fluorescence microscope.

### Western blotting analysis

After treatment, cells were washed with PBS for 3 times and lysed in lysis butter (1% TritonX-100, 0.015 M NaCl, 10 mM Tris–HCl, 1 mM EDTA, 1 mM PMSF, 10 lg/mL of each leupeptin and pepstain A). The protein concentrations were measured with a BCA kit. The cell lysates were solubilized in SDS sample buffer, separated by sodium dodecyl sulfate-polyacrylamide gel electrophoresis (SDS-PAGE), and transferred to a polyvinylidene fluoride (PVDF) membranes. The membrane was blocked with blocking buffer (5% nonfat dry milk) overnight at 4°C. After that, the membrane was incubated with the indicated primary antibody with OPN(1∶500), occludin(1∶250), MLCK(1∶500), pMLC(1∶500), MLC(1∶1000), pJNK(1∶500), JNK(1∶1000), and β-actin(1∶1000) respectively and followed by the appropriate horseradish peroxidase(HRP)-conjugated secondary antibody, and visualized with enhanced chemiluminescence using hydrogen peroxide and luminol as substrate with Kodak X-AR film. The images were scanned using a ScanPrisa1240 OUT (Acer, China). Western blots data were quantified using Quantity One software.

### Statistical analysis

Three or more separate experiments were performed for each experiment. Statistical analysis was performed by Student’s t-test or ANOVE. Data are presented as means ± standard deviation. Statistical significance was defined as *p* value less than 0.05.

## Results

### Effect of melatonin on viability in A549 cells

To determine the effect of melatonin on cell viability, A549 cells were treated with different concentrations of melatonin (0.1, 0.5, 0.75, 1.0, 2.5, 5.0 mmol/L) for 3 d. Viability was assessed using the MTT assay. The results showed that melatonin inhibited the proliferation of A549 cells in a concentration-dependent manner, compared with the control group. The inhibition rates were 17.50%, 23.53%, 29.00%, 38.78%, 72.99% and 84.15%, respectively (Table 1). The IC 50 of melatonin is 1.864 mmol/L.

**Table 1:** The effect of different concentrations of MLT on the viability of A549 cells in 3 day.

Group	OD570 nm ( 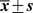 )	Inhibition rate (%)	IC50 (mmol/L)
DMSO	0.67±0.04	–	1.864
MLT (mmol/L)			
0.10	0.55±0.05	17.50*	
0.50	0.51±0.02	23.53*	
0.75	0.47±0.02	29.00*	
1.00	0.41±0.03	38.78*	
2.50	0.18±0.02	72.99*	
5.00	0.10±0.01	84.15*	

Compared with DMSO control group, *P<0.05.

### Effect of melatonin on migration in A549 cells

**Figure 1:**
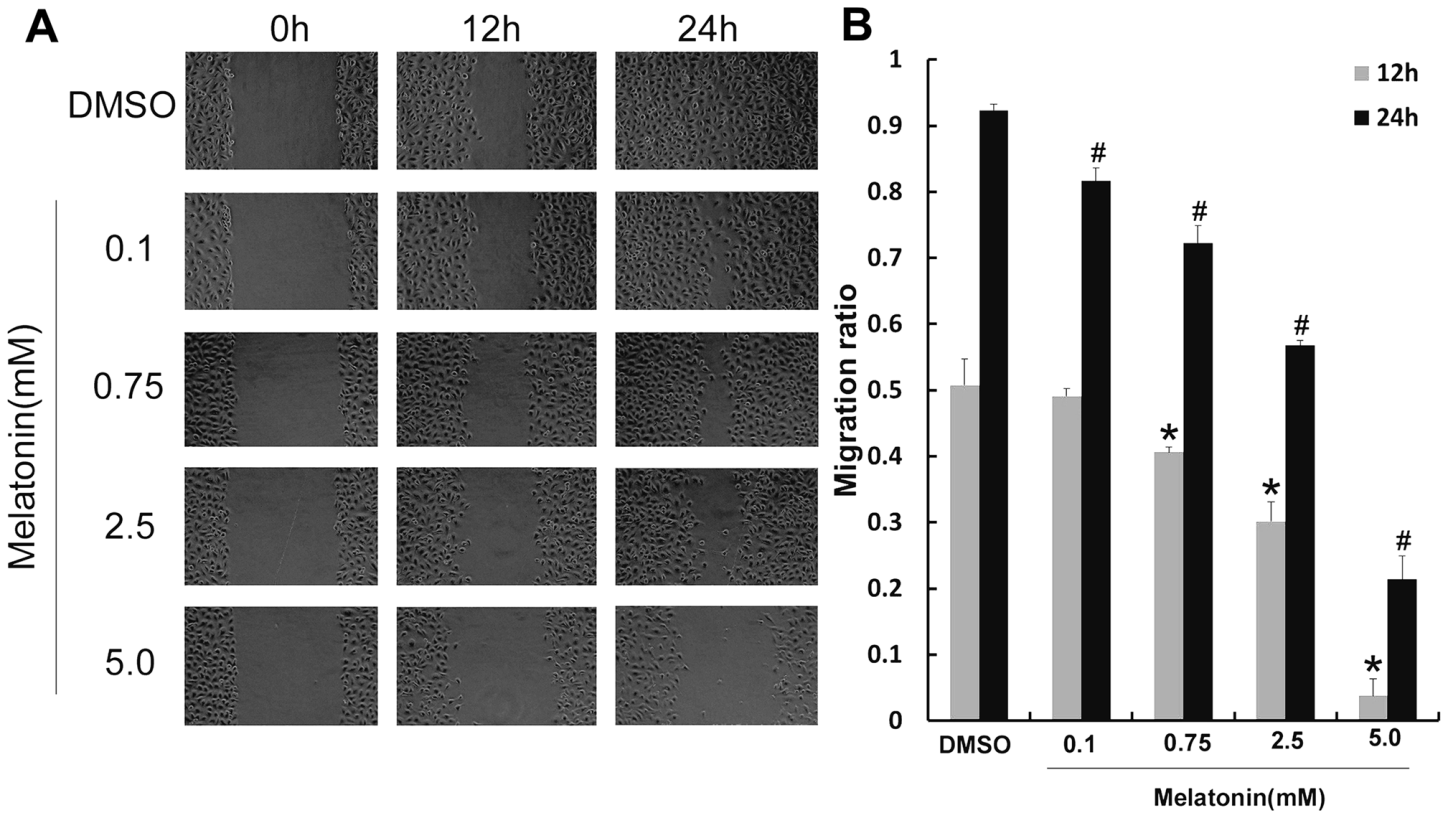
Melatonin inhibits the migration of A549 cells. (A) The migration of A549 cells at 0.1, 0.75, 2.5, 5.0 mmol/L melatonin groups respectively when A549 cells were treated for 0 h, 12 h and 24 h. (B) Analysis of migration rate (%), compared with control group (DMSO): *P<0.05, ^#^P<0.05.

**Figure 2:**
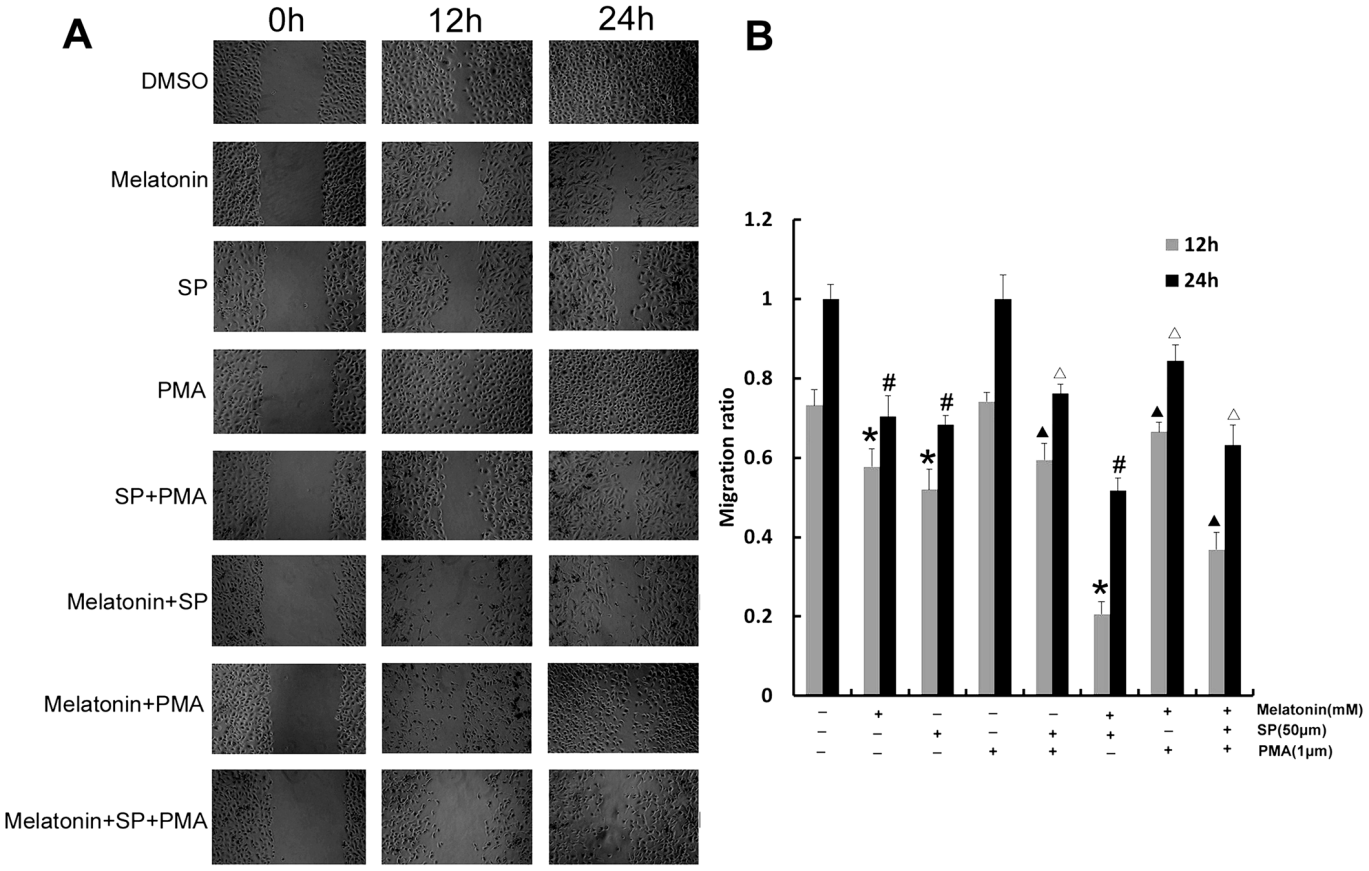
The effect of melatonin, SP600125 and PMA on migration of A549 cells. (A) The effect of melatonin, SP600125 and PMA on migration of A549 cells after 0 h, 12 h and 24 h. (B) Analysis of migration rate, compared with control group: *P<0.05, ^#^P<0.05; compared with PMA group: ^▴^P<0.05, ^△^P<0.05.

To investigate the effect of melatonin on migration, A549 cells were treated with different concentrations of melatonin. Migration of A549 cells were inhibited by melatonin in a concentration-dependent manner (Fig. 1). We then used pharmacological inhibitors and activator to determine the role of JNK in the migration of A549 cells. The A549 cells were treated with SP600125 (JNK inhibitor) and PMA (MAPK activator) for 3 d. The results showed that SP600125 significantly inhibited the migration of A549 cells and PMA has no obvious effect on the migration of A549 cells compared with the control while PMA decreased the effects of Melatonin and SP600125 on migration of A549 cells (p<0.05) (Fig. 2).

### Melatonin up-regulates the expression of occludin and enhances occludin to locate on the cell surface, and down-regulates the expression of OPN, MLCK in A549 cells, which is partly through the JNK/MAPK signaling pathway

**Figure 3:**
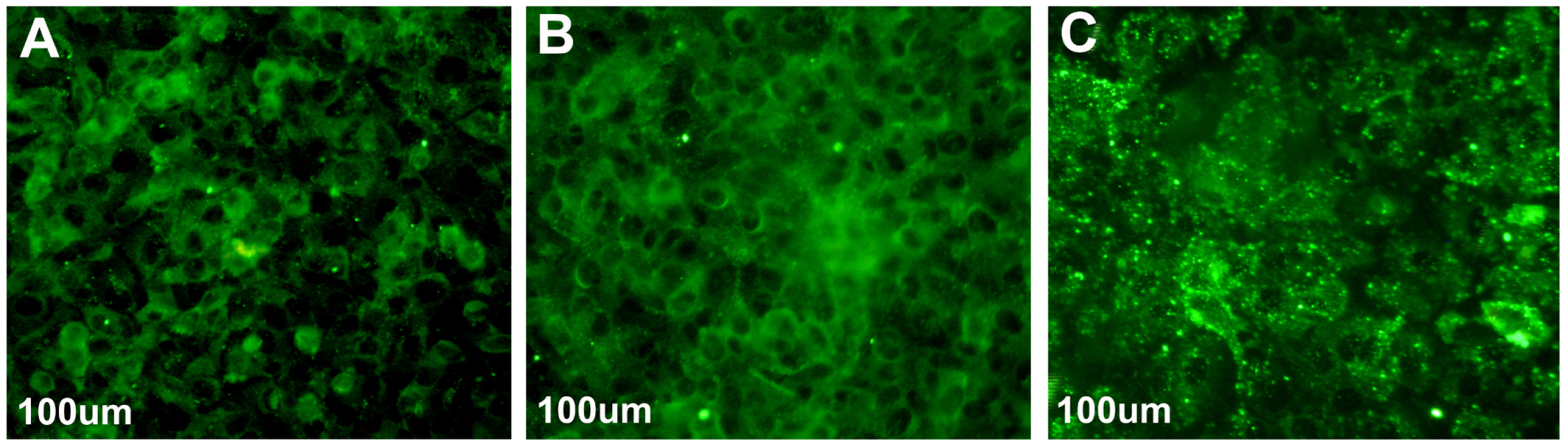
Immunofluorescence detection of tight junction correlated protein occludin in A549 cells. (A) DMSO control. (B) melatonin (0.1 mmol/L). (C) melatonin (2.0 mmol/L).

**Figure 4:**
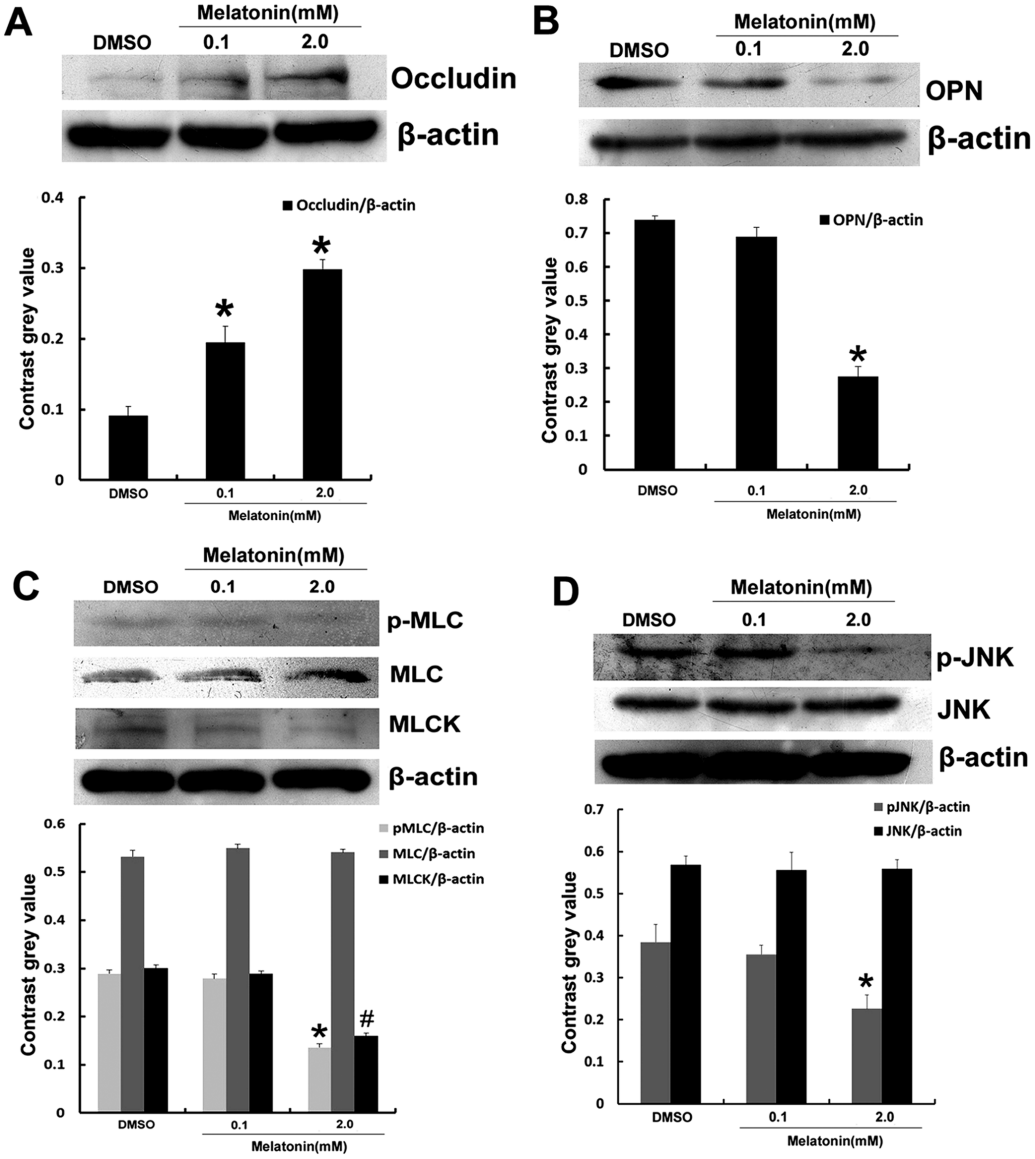
Effect of melatonin on the expression of occludin, OPN, MLCK and phosphorylation of MLC, JNK. (A) occludin, (B) OPN, (C) MLC and MLCK, (D) JNK. Results are presented as mean ± SD of three independent experiments. *P<0.05, in comparison to control group.

Tight junction correlated protein occludin was detected by immunofluorescence in A549 cells. The results showed that there was no occludin accumulation on the A549 cells surface, but occludin started to locate on the cell surface when A549 cells was treated with melatonin at the concentration of 0.1 mmol/L. Occludin located on the cell surface was obviously increased when treated with melatonin at the concentration of 2.0 mmol/L (Fig. 3). The effect of melatonin on the expression of proteins related with migration was determined using western blots analysis in A549 cells. After the cells were treated with different concentrations of melatonin for 3 d, the results revealed that melatonin (2.0 mmol/L) enhanced the expression of occludin (p<0.05) (Fig. 4 A), reduced the expression of OPN (Fig. 4 B), MLCK and phosphorylation of MLC (Fig. 4 C), JNK (Fig. 4 D).

**Figure 5:**
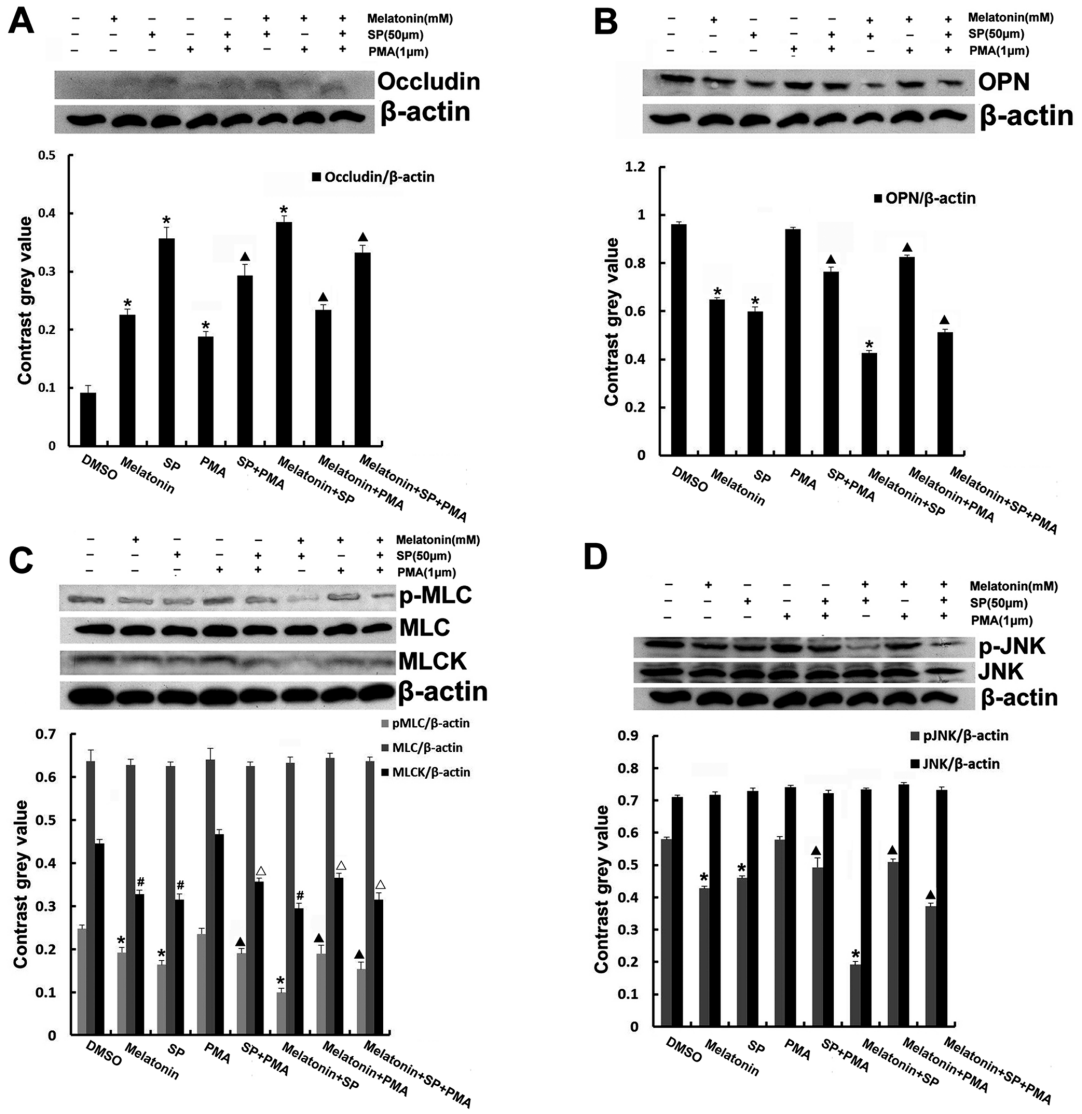
The Effect of melatonin, SP600125 and PMA on the expression of occludin, OPN, MLCK and phosphorylation of MLC, JNK. (A) occludin, (B) OPN, (C) MLC and MLCK, (D) JNK. Results are presented as mean ± SD of three independent experiments. *P<0.05,^ #^P<0.05, in comparison to control; ^▴^P<0.05, ^△^P<0.05, in comparison to PMA group.

After that, we examined whether the expression of related proteins are associated with JNK/MAPK. In later western blots experiment, we then used pharmacological inhibitors and activator to determine the role of JNK in the expression of related proteins in A549 cells, the cells were treated with melatonin, SP600125, and PMA for 3 d. The phosphorylation status of JNK was decreased. The expression level of OPN, MLCK, and phosphorylation of MLC were down-regulated, while the expression of occludin was up-regulated when cells were exposed to melatonin and SP600125 compared with control group (p<0.05). The expression level of OPN, MLCK, phosphorylation of MLC, JNK were lower in melatonin plus SP600125 group while the expression level of occludin was increased (p<0.05) (Fig. 5).

## Discussion

Cell migration is a biological process that contributes crucially to a variety of physiological functions, such as wound healing and the inflammatory reaction. Moreover, cell migration is also responsible for the malignance of cancer as it allows tumor cells to invade the surrounding tissues, thereby forming metastases [4]. Melatonin can inhibit tumor cell proliferation, selectively blocking the signal transduction of tumor cells, and destroying its autonomous growth, which has become a research hot point [32], [44]–[45]. Recently, several studies have shown that melatonin can also regulate microtubule and microfilament structure formation and inhibit tumor cell invasion and metastasis process [46].

In our studies, we demonstrate, for the first time, the effect of melatonin on the migration of human lung adenocarcinoma A549 cells and observed an association between JNK/MAPK pathway and the expression of tight junction (TJ) related proteins occludin, OPN and MLCK. Some studies have found that melatonin affects cell cycle as it prolongs the cell cycle of cancer cells and delays tumor cells to enter mitosis, thus making cancer cell proliferation restrained. Melatonin inhibits cell proliferation via downstream cyclin E from cells entering S phase [47]–[48]. Our current data show that melatonin could inhibit the viability of A549 cells in a concentration-dependent manner, compared with control group. The melatonin may inhibit A549 cell proliferation and play an important role in the inhibition of tumor progression.

Recently, studies found that melatonin could convert cell subtype of microtubules and make the invasive cells into non-migrating cells with stationary microtubules subtypes in breast cancer MCF-7 cells [46]. Our results showed that migration distance shortened obviously when melatonin was used to treat A549 cells for 24 h and demonstrated that melatonin reduced the migration of A549 cell.

Occludin is the main functional regulatory protein in TJ and is thought to be the most sensitive and reliable sign of tight junction structure. The expression and dysfunction of occludin proteins is associated with the development and metastasis of tumor [49]–[50]. We observed that the expression of occludin was significantly increased in A549 cells treated with melatonin at the concentration of 0.1 mmol/L and 2.0 mmol/L. Immunofluorescence result revealed that melatonin promoted occludin to locate on the cell surface. The TJ leaks of human brain tumors Microvessels are responsible for cerebral edema in certain types of brain cancer, which is an excellent example of down-regulation of occludin in cancer [51]–[52]. We speculated that melatonin promoted occludin proteins to move and position on the cell surface and enhanced the formation of close connection between cells, which have the effect of inhibiting A549 cell metastasis.

OPN has repeatedly been shown to be present at high levels in the circulation of patients with metastatic cancers [53]–[57] and increased metastatic potential [58]–[60], thus make it relevant in the context of studying its expression in the perspective of metastasis. Both activated myosin II and its activator MLCK are enriched in lamellar protrusive structures in several cell types during migration [61]. MLCK activation and expression have been found to be positively related with metastatic propensity [62]–[63]. Our results demonstrated that melatonin significantly suppressed the JNK/MAPK pathway in A549 cells. To determine the mechanism, we used PMA and SP600125 to respectively activate and inhibit MAPK/JNK signaling in A549 cells. Our results showed that SP600125 significantly inhibited the migration of A549 cells. Melatonin and SP600125 inhibited the relative migration rate of groups in PMA-stimulated groups. The expression of OPN and MLCK, the phosphorylated of MLC were down-regulated while the expression level of occludin was up-regulated when cells were exposed to SP600125. Melatonin and SP600125 inhibited the expression of MLCK, OPN and phosphorylated of JNK, MLC, and enhance the expression of occludin in PMA-stimulated groups. In accordance with our data, therefore, the anti-migration effect of melatonin is associated with its inhibition of JNK/MAPK pathway and regulation of the expression of occludin, OPN, and MLCK.

Our present results show that melatonin plays an important role in inhibiting the proliferation and migration of A549 cells. Occludin, OPN, and MLCK contribute to the migration of A549 cells involving JNK/MAPK pathway. Our findings support the potential application of melatonin in the treatment of lung cancer. Due to the migration of tumor cells and the formation of tumor metastasis is an extremely complex process which involves multiple steps and many factors, the comprehensive mechanism of melatonin inhibits tumor cell migration still needs further research.

## References

[1] Siegel R, Naishadham D, Jemal A (2012) Cancer statistics, 2012. A Cancer Journal for Clinicians 62: 10–29. 10.3322/caac.20138 22237781

[2] Petty RD, Nicolson MC, Kerr KM, Collie-Duguid E, Murray GI (2004) Gene expression profiling in non-small cell lung cancer: from molecular mechanisms to clinical application. Clinical Cancer Research 10: 3237–3248. 15161676 10.1158/1078-0432.CCR-03-0503

[3] Dziadziuszko R, Hirsch FR (2008) Advances in genomic and proteomic studies of non-small-cell lung cancer: clinical and translational research perspective. Clinical Lung Cancer 9: 78–84. 18501093 10.3816/CLC.2008.n.012

[4] Weight B, Peterse JL, Van’t Veer LJ (2005) Breast cancer metastasis: markers and models. Nature Reviews Cancer 5: 591–602. 16056258 10.1038/nrc1670

[5] Vicente-Manzanares M, Zareno J, Whitmore L, Choi CK, Horwitz AF (2007) Regulation of protrusion, adhesion dynamics, and polarity by myosins IIA and IIB in migration cells. The Journal of Cell Biology 176: 573–580. 17312025 10.1083/jcb.200612043 PMC2064016

[6] Tuck AB, Hota C, Wilson SM, Chambers AF (2003) Osteopontin-induced migration of human mammary epithelial cells involves activation of EGF receptor and multiple signal transduction pathways. Oncogene 22: 1198–1205. 12606946 10.1038/sj.onc.1206209

[7] Chew TL, Wolf WA, Gallagher PJ, Matsumura F, Chisholm RL (2002) A Fluorescent resonant energy transfer based biosensor reveals transient and regional myosin light chain kinasdxce activation in lamella and cleavage furrows. The Journal of Cell Biology 156: 543–553. 11815633 10.1083/jcb.200110161 PMC2173328

[8] Kishi H, Mikawa T, Seto M, Sasaki Y, Kanayasu-Toyoda T, et al (2000) Stable transfectants of smooth muscle cell line lacking the expression of myosin light chain kinase and their characterization with respect to the actomyosin system. The Journal of Biological Chemistry 275: 1414–1420. 10625693 10.1074/jbc.275.2.1414

[9] Sodek J, Ganss B, McKee MD (2000) Osteopontin. Critical Reviews in Oral Biology&Medicine 11: 279–303. 11021631 10.1177/10454411000110030101

[10] Furuse M, Hirase T, Itoh M, Nagafuchi A, Yonemura S, et al (1993) Occludin: a novel integral membrane protein localizing at tight junctions. The Journal of Cell Biology 123: 1777–1788. 8276896 10.1083/jcb.123.6.1777 PMC2290891

[11] Ando-Akatsuka Y, Saitou M, Hirase T, Kishi M, Sakakibara A, et al (1996) Interspecies diversity of the occludin sequence: cDNA cloning of human, mouse, dog, and rat-kangaroo homologues. The Journal of Cell Biology 133: 43–47. 8601611 10.1083/jcb.133.1.43 PMC2120780

[12] Fujimoto K (1995) Freeze-fracture replica electron microscopy combined with SDS digestion for cytochemical labeling of integral membrane proteins. Application to the immunogold labeling of intercellular junctional complexes. Journal of Cell Science 108: 3443–3449. 8586656 10.1242/jcs.108.11.3443

[13] Dhawan P, Singh AB, Deane NG, No Y, Shiou SR, et al (2005) Claudin-1 regulates cellular transformation and metastatic behavior in colon cancer. Journal of Clinical Investigation 115: 1765–1776. 15965503 10.1172/JCI24543 PMC1150288

[14] Hoover KB, Liao SY, Bryant PJ (1998) Loss of the tight junction MAGUK ZO-1 in breast cancer: relationship to glandular differentiation and loss of heterozygosity. The American Journal of Pathology 153: 1767–1773. 9846967 10.1016/S0002-9440(10)65691-X PMC1866327

[15] Tobioka H, Tokunaga Y, Isomura H, Kokai Y, Yamaguchi J, et al (2004) Expression of occludin, a tight-junction associated protein, in human lung carcinomas. Virchows Arch 445: 472–476. 15232740 10.1007/s00428-004-1054-9

[16] Gonzalez-Mariscal L, Tapia R, Chamorro D (2008) Crosstalk of tight junction components with signaling pathways. Biochimica et Biophysica Acta 1778: 729–756. 17950242 10.1016/j.bbamem.2007.08.018

[17] Chang L, Karin M (2001) Mammalian MAP kinase signaling cascades. Nature 410: 37–40. 11242034 10.1038/35065000

[18] Yang R, Piperdi S, Gorlick R (2008) Activation of the RAF/mitogen-activated protein/extracellular signal-regulated kinase kinase/extracellular signal-regulated kinase pathway mediates apoptosis induced by chelerythrine in osteosarcoma. Clinical Cancer Research 14: 6396–6404. 18927278 10.1158/1078-0432.CCR-07-5113

[19] Ip YT, Davis RJ (1998) Signal transduction by the c-Jun N-terminal kinase (JNK)-from inflammation to development. Current Opinion in Cell Biology 10: 205–219. 9561845 10.1016/s0955-0674(98)80143-9

[20] Bogoyevitch MA, Kobe B (2006) Uses for JNK: the many and varied substrates of the c-Jun N-terminal kinases. Microbiology and Molecular Biology Reviews 70: 1061–1095. 17158707 10.1128/MMBR.00025-06 PMC1698509

[21] Dominguez-Rodriguez A, Breu-Gonzalez P (2011) Melatonin: still a forgotten antioxidant. International Journal of Cardiology 149: 382. 21414673 10.1016/j.ijcard.2011.02.070

[22] Korkmaz A, Reiter RJ, Topal T, Manchester LC, Oter S, et al (2009) Melatonin: an established antioxidant worthy of use in clinical trials. Molecular Medicine 15: 43–50. 19011689 10.2119/molmed.2008.00117 PMC2582546

[23] Galano A, Tan DX, Reiter RJ (2011) Melatonin as a naturalally against oxidative stress: a physicochemical examination. Journal of Pineal Research 51: 1–16. 21752095 10.1111/j.1600-079X.2011.00916.x

[24] Bonnefont-Rousselot D, Collin F, Jore D, Gardès-Albert M (2011) Reaction mechanism of melatonin oxidation by reactive oxygen species in vitro. Journal of Pineal Research 50: 328–335. 21244479 10.1111/j.1600-079X.2010.00847.x

[25] Reiter RJ, Tan DX, Poeggeler B, Menendez-Pelaez, Chen L, et al (1994) Melatonin as a free radical scavenger: implications for aging and age-related diseases. Annals of The New York Academy of Sciences 719: 1–12. 8010585 10.1111/j.1749-6632.1994.tb56817.x

[26] Tan DX, Chen LD, Poeggeler B, Manchester LC, Reiter RJ, et al (1993) Melatonin: a potent endogenous hydroxyl radical scavenger. Endocrine Journal 1: 57–60.

[27] Hill SM, Blask DE, Xiang S, Yuan L, Mao L, et al (2011) Melatonin and associated signaling pathways that control normal breast epithelium and breast cancer. Journal of Mammary Gland Biology and Neoplasia 16: 235–245. 21773809 10.1007/s10911-011-9222-4

[28] Messina G, Lissoni P, Marchiori P, Bartolacelli E, Brivio F, et al (2010) Enhancement of the efficacy of cancer chemotherapy by the pineal hormone melatonin and its relation with the psychospiritual status of cancer patients. Journal of Research in Medical Sciences 15: 225–228. 21526086 PMC3082810

[29] Padillo FJ, Ruiz-Rabelo JF, Cruz A, Perea MD, Tasset I, et al (2010) Melatonin and celecoxib improve the outcomes in hamsters with experimental pancreatic cancer. Journal of Pineal Research 49: 264–270. 20626589 10.1111/j.1600-079X.2010.00791.x

[30] Grant SG, Melan MA, Latimer JJ, Witt-Enderby PA (2009) Melatonin and breast cancer: cellular mechanisms, clinical studies and future perspectives. Expert Reviews in Molecular Medicine 11: e5. 19193248 10.1017/S1462399409000982 PMC4301735

[31] Srinivasan V, Spence DW, Pandi-Perumal SR, Trakht I, Cardinali DP, et al (2008) Therapeutic actions of melatonin in cancer: possible mechanisms. Integrative Cancer Therapies 7: 189–203. 18815150 10.1177/1534735408322846

[32] Garcia-Navarro A, Gonzalez-Puga C, Escames G, López LC, López A, et al (2007) Cellular mechanisms involved in the melatonin inhibition of HT-29 human colon cancer cell proliferation in culture. Journal of Pineal Research 43: 195–205. 17645698 10.1111/j.1600-079X.2007.00463.x

[33] Jung-Hynes B, Reiter RJ, Ahmad N (2010) Sirtuins, melatonin and circadian rhythms: building a bridge between aging and cancer. Journal of Pineal Research 48: 9–19. 20025641 10.1111/j.1600-079X.2009.00729.x PMC2948667

[34] Gonzalez A, Del Castillo-Vaquero A, Miro-Moran A, Tapia JA, Salido GM, et al (2011) Melatonin reduces pancreatic tumor cell viability by altering mitochondrial physiology. Journal of Pineal Research 50: 250–260. 21118301 10.1111/j.1600-079X.2010.00834.x

[35] Um HJ, Park JW, Kwon TK (2011) Melatonin sensitizes Caki renal cancer cells to kahweol-induced apoptosis through CHOP mediated up-regulation of PUMA. Journal of Pineal Research 50: 359–366. 21244481 10.1111/j.1600-079X.2010.00851.x

[36] Mao L, Yuan L, Slakey LM, Jones FE, Burow ME, et al (2010) Inhibition of breast cancer cell invasion by melatonin is mediated through regulation of the p38 mitogen-activated protein kinase signaling pathway. Breast Cancer Research 12: R107. 21167057 10.1186/bcr2794 PMC3046452

[37] Proietti S, Cucina A, D’Anselmi F, Dinicola S, Pasqualato A, et al (2011) Melatonin and vitamin D3 synergistically down-regulate Akt and MDM2 leading to TGFbeta-1-dependent growth inhibition of breast cancer cells. Journal of Pineal Research 50: 150–158. 21091766 10.1111/j.1600-079X.2010.00824.x

[38] Martínez-Campa CM, Alonso-González C, Mediavilla D, Cos S, González A, et al (2008) Melatonin down-regulates hTERT expression induced by either natural estrogens (17beta-estradiol) or metalloestrogens (cadmium) in MCF-7 human breast cancer cells. Cancer Letters 268: 272–277. 18479810 10.1016/j.canlet.2008.04.001

[39] Dai M, Cui P, Yu M, Han J, Li H, et al (2008) Melatonin modulates the expression of VEGF and HIF-1 alpha induced by CoCl2 in cultured cancer cells. Journal of Pineal Research 44: 121–126. 18289162 10.1111/j.1600-079X.2007.00498.x

[40] Cos S, Fernández R, Guezmes A, Sánchez-Barceló EJ (1998) Influence of melatonin on invasive and metastatic properties of MCF-7 human breast cancer cells. Cancer research 58: 4383–4390. 9766668

[41] Ortiz-Lopez L, Morales-Mulia S, Ramirez-Rodriguez G, Benítez-King G (2009) ROCK-regulated cytoskeletal dynamics participate in the inhibitory effect of melatonin on cancer cell migration. Journal of Pineal Research 46: 15–21. 18482340 10.1111/j.1600-079X.2008.00600.x

[42] Ramirez-Rodriguez G, Ortiz-Lopez L, Benitez-King G (2007) Melatonin increases stress fibers and focal adhesions in MDCK cells: participation of Rho-associated kinase and protein kinase C. Journal of Pineal Research. 42: 180–190. 10.1111/j.1600-079X.2006.00404.x 17286751

[43] Bellon A, Ortiz-Lopez L, Ramirez-Rodriguez G, Antón-Tay F, Benitez-King G (2007) Melatonin induces neuritogenesis at early stages in N1E-115 cells through actin rearrangements via activation of protein kinase C and Rho-associated kinase. Journal of Pineal Research 42: 214–221. 17349018 10.1111/j.1600-079X.2006.00408.x

[44] Tam CW, Mo CW, Yao KM, Stephen YW, Shiu SY (2007) Signaling mechanisms of melatonin in anti-proliferation of hormone-refractory 22Rv1 human prostate cancer cells: implications for prostate cancer chemoprevention. Journal of Pineal Research 42: 191–202. 17286752 10.1111/j.1600-079X.2006.00406.x

[45] AL-Shenbel IF, Shidata HR, Sampalis J, Jothy S (1993) Prognostic significance of proliferating cell nuclear antigen expression colorectal cancer. Cancer 71: 1954–1959. 8095176 10.1002/1097-0142(19930315)71:6<1954::aid-cncr2820710605>3.0.co;2-#

[46] Bnítez-King G, Soto-Vega E, Ramírez-Rodriguez G (2009) Melatonin modulates microfilament phenotypes in epithelial cells: implications for adhesion and inhibition of cancer cell migration. Histology and Histopathology 24: 789–799. 19337976 10.14670/HH-24.789

[47] Fornas O, Mato ME, Webb SM (2000) Anti-proliferative effect and cell cycle modulation by melatonin on GH (3) cells. Hormone Research in Paediatrics 53: 251–255. 10.1159/000023575 11150887

[48] Mediavilla MD, Cos S, Sánchez-Barceló EJ (1999) Melatonin increases p53 and p21WAF1 expression in MCF-7 human breast cancer cells in vitro. Life Sciences 65: 415–420. 10421427 10.1016/s0024-3205(99)00262-3

[49] Tobioka H, Isomura H, Kokai Y, Tokunaga Y, Yamaguchi J, et al (2004) Occludin expression decreases with the progression of human endometrial carcinoma. Human pathology 35: 159–164. 14991532 10.1016/j.humpath.2003.09.013

[50] Kimura Y, Shiozaki H, Hirao M, Maeno Y, Doki Y, et al (1997) Expression of occludin, tight junction associated protein, in human digestive tract. American Journal of Pathology 151: 45–54. 9212730 PMC1857944

[51] Davies DC (2002) Blood-brain barrier breakdown in septic encephalopathy and brain tumor. Journal of Anatomy 200: 639–646. 12162731 10.1046/j.1469-7580.2002.00065.x PMC1570752

[52] Papadopoulos MC, Saadoun S, Woodrow CJ, Davies DC, Costa-Martins P, et al (2001) Occludin expression in microvessels of neoplastic and non- neoplastic human brain. Neuropathology and Applied Neurobiology 27: 384–395. 11679090 10.1046/j.0305-1846.2001.00341.x

[53] Bramwell VH, Doig GS, Tuck AB, Wilson SM, Tonkin KS, et al (2006) Serial plasma osteopontin levels have prognostic value in metastatic breast cancer. Clinical Cancer Research 12: 3337–3343. 16740755 10.1158/1078-0432.CCR-05-2354

[54] Hotte SJ, Winquist EW, Stitt L, Chambers AF (2002) Plasma Osteopontin. Cancer 95: 506–512. 12209742 10.1002/cncr.10709

[55] Fedarko NS, Jain A, Karadag A, Van Eman MR, Fisher LW (2001) Elevated serum bone sialoprotein and osteopontin in colon, breast, prostate, and lung cancer. Clinical Cancer Research 7: 4060–4066. 11751502

[56] Kim JH, Skates SJ, Uede T, Wong KK, Schorge JO, et al (2002) Osteopontin as a potential diagnostic biomarker for ovarian cancer. The Journal of the American Medical Association 287: 1671–1679. 11926891 10.1001/jama.287.13.1671

[57] Bramwell VH, Tuck AB, Wilson SM, Stitt LW, Cherian AK, et al (2005) Expression of osteopontin and HGF/met in adult soft tissue tumors. Cancer Biology and Therapy 4: 1336–1341. 16258259 10.4161/cbt.4.12.2166

[58] Chambers AF, Behrend EI, Wilson SM, Denhardt DT (1992) Induction of expression of osteopontin (osteopontin; secreted phosphoprotein) in metastatic, ras-transformed NIH 3T3 cells. Anticancer Research 12: 43–47. 1567180

[59] Oates AJ, Barraclough R, Rudland PS (1996) The identification of osteopontin as a metastasis-related gene product in a rodent mammary tumour model. Oncogene 13: 97–104. 8700559

[60] Ramankulov A, Lein M, Kristiansen G, Loening SA, Jung K (2007) Plasma osteopontin in comparison with bone markers as indicator of bone metastasis and survival outcome in patients with prostate cancer. The Prostate 67: 330–340. 17192877 10.1002/pros.20540

[61] Kolega L (2003) Asymmetric distribution of myosin IIB in migration endothelial cells is regulated by a rho-dependent kinase and contributes to tail retraction. Molecular Biology of the Cell 14: 4745–4757. 12960430 10.1091/mbc.E03-04-0205 PMC284780

[62] Tohtong R, Phattarasakul K, Jiraviriyakul A, Sutthiphongchai T (2003) Dependence of metastatic cancer cell invasion on MLCK-catalyzed phosphorylation of myosin regulatory light chain. Prostate Cancer and Prostatic Diseases 6: 212–216. 12970723 10.1038/sj.pcan.4500663

[63] Minamiya Y, Nakagawa T, Saito H, Matsuzaki I, Taguchi K, et al (2005) Increased expression of myosin light chain kinase mRNA is related to metastasis in non-small cell lung cancer. Tumour Biology 26: 153–157. 15970650 10.1159/000086487

